# Molecular Detection of *Bartonella* spp. in Rodents in Chernobyl Exclusion Zone, Ukraine

**DOI:** 10.1007/s11686-020-00276-1

**Published:** 2020-09-19

**Authors:** Tomasz Szewczyk, Joanna Werszko, Kateryna Slivinska, Zdzisław Laskowski, Grzegorz Karbowiak

**Affiliations:** 1 W. Stefański Institute of Parasitology, Polish Academy of Sciences, Twarda 51/55, 00-818 Warsaw, Poland; 2 I. I. Schmalhausen Institute of Zoology of National Academy of Sciences of Ukraine, Vul. B. Khmelnytskogo, 15, Kiev, 01030 Ukraine

**Keywords:** *Bartonella* spp., Rodents, Ukraine, Chernobyl Exclusion Zone

## Abstract

**Purpose:**

Bacteria of the genus *Bartonella* are obligate parasites of vertebrates. Their distribution range covers almost the entire world, from the Americas to Europe and Asia. Many *Bartonella* species use rodents as reservoirs, and while much is known about *Bartonella* infection of rodents in central Europe, its extent is poorly understood in Eastern Europe.

**Methods:**

The present study examines five rodent species (*Apodemus flavicollis*, *Myodes glareolus*, *Microtus arvalis*, *Apodemus agrarius*, *Apodemus sylvaticus*) in the Chernobyl Exclusion Zone in Ukraine. Total of 36 small mammals were captured in September 2017.

**Results:**

The overall prevalence of *Bartonella* spp. was 38.9% (14/36) in rodents. Obtained four sequences from *Apodemus flavicollis*, were identical to *Bartonella grahamii* and *B. taylorii*.

**Conclusion:**

This is the first report to confirm the presence of *Bartonella* spp. in rodents in the Chernobyl Exclusion Zone, Ukraine by molecular methods. The sequences show similarity to *Bartonella* strains occurring in Europe.

## Introduction

The Chernobyl Exclusion Zone (CEZ) was established around the site of the 1986 disaster at the Chernobyl nuclear power plant. The area around the nuclear power plant was irradiated. Since this time, the inhabitants were resettled to another part of Ukraine and wild animals were allowed to return to the abandoned by people areas. The direct consequences of radioactive pollution, as well as the evacuation of the human population and cessation of agriculture and forest management resulted in significant ecological changes, such as the spontaneous restitution of the original natural habitats, the plant and animal populations, and their interdependences. The wild mammal population is now numerous and strong. Many aspects of the consequences of radionuclide contamination of wild animals have been studied during the short time since the catastrophe. For instance, Frantsevich [13] examines radionuclide accumulation in the soil and by different species of animals, Møller and Mousseau [29] report the effects of radiation on cytogenetics and mutation, and Møller et al. [30] examine the frequency of abnormalities potentially caused by such pollution. The increase of micromammalian parasite complexes and their biodiversity have also been examined [25, 34]. However, the parasite fauna of wild animals, including those of the small rodents in Chernobyl Exclusion Zone, is poorly understood, as are the observations of arthropod-borne pathogens [19]. The genus *Bartonella* comprises small, Gram-negative bacteria which act as obligate intracellular parasites of vertebrates. More than 30 species, as well as three subspecies, have been described [3, 7, 15]. Many of *Bartonella* species have been associated with emerging diseases, the symptoms of which have a broad spectrum of clinical syndromes ranging from cat-scratch diseases to potentially fatal conditions, such as endocarditis [1, 8, 9, 20–22, 43, 44]. Some *Bartonella* species, including *Bartonella elizabethae*, *Bartonella tribocorum*, *Bartonella grahamii*, *Bartonella vinsonii* subsp. *arupensis* and *Bartonella washoensis*, are causative agents of human infections [9, 21, 22, 43], and some researchers consider these bacteria to be host-specific, e.g. *B. elizabethae* and *B. tribocorum* are specific to *Rattus* spp. [3]. *Bartonella* species are transmitted by blood-sucking arthropods, such as sandflies, lice, fleas [6, 16, 31, 39]; in addition, *Bartonella* spp. have also been found in keds: *Lipoptena cervi*, *Melophagus ovinus* [11, 12, 40]. *Bartonella* species have also been common in selected rodents, ruminants and carnivores; the prevalence of infection in these animals group may be very high [6, 18] and strongly depends on the season [18]. Many animal-associated *Bartonella* species have been described and can be a threat to people. A high prevalence of *Bartonella* spp. has been observed in rodents in Asia 51%, in America 42% and in Africa 29% [2, 23, 28, 42]. Their prevalence in rodents in Central Europe ranges from 3.3 to 65.8% [18, 24, 26, 37, 41]. In European rodents, four species have been observed to be the most widespread: *Bartonella grahamii*, *B. taylori*, *B. birtlesii* and *B. rochalimae*. In Central Europe, the prevalence of *Bartonella* infection in small rodents is quite well known. Studies report a 65.8% prevalence in Germany, 9.0% in Slovenia, 3.3% in Croatia, 23.7% in Lithuania and 28% in Poland [18, 24, 26, 37, 41]. Although some reports exist of *Bartonella* infection in rodents in Eastern Europe, our understanding of their distribution remains incomplete. Therefore, the aim of this study was to analyse the occurrence of *Bartonella* species in rodents living near the abandoned city in the Chernobyl Exclusion Zone, Ukraine.

## Materials and Methods

### Study Area

The CEZ is located c. 200 km N of Kiev, Ukraine (51° N; 30.005° E), 123 m above sea level. This zone cover an area of 2600 km^2^ and falls entirely within the Polesie Lowland, Russian Plain. The climate in the CEZ is humid, with relatively mild winters and warm summers. The mean annual temperature is 5–7 °C, with a mean temperature in July of 18 °C (max. 32 °C), and in January of − 6 °C (min. − 25 °C). The annual precipitation ranges from 550 to 750 mm. The snow cover lasts on average c. 50 days per year. The mean depth of the snow cover is 12–13 cm [5].

The CEZ is surrounded by metal fencing 2 m high. Approximately c. 50 peasants also are still living in the CEZ; only few other persons have constant access to this area. Before the nuclear disaster, the CEZ consisted of farmland with patchy areas of forest. Currently, ca. 60% of the area is covered with forests, 50% of which is pine forest and remaining consist of abandoned arable grounds, meadows, pastures and human settlements.

### Data Collection

Rodents were collected to the 3.2 km west of Chernobyl city (51°17′0′′ N; 30°13′2′′ E) in September 2017, using Sherman live-traps. The traps were checked two times per day. The captured mammals were transported in their traps to the laboratory for examination. The autopsies were carried out under terminal isoflurane anesthesia. Blood was collected for smear samples and spleen samples were taken. These were stored at − 20 °C and transported to the laboratory. The following animals were captured, in order of quantity: bank vole (*Myodes glareolus*)—13 animals, 4 m/9f (36.1% of total), yellow-necked mice (*Apodemus flavicollis*)—12 animals, 4 m/8f (33.3%), and striped field mice (*Apodemus agrarius*)—seven animals, 4 m/3f (19.44%). Rarer species included the common vole (*Microtus arvalis*)—three animals, 2 m/1f (8.3%), and one male wood mouse (*Apodemus sylvaticus*).

### DNA Isolation and PCR Amplification

The DNA was isolated from spleen using the AX Tissue Mini Kit (A&A Biotechnology, Gdynia, Poland) according to the manufacturer’s protocol. For the PCR test, the primers rpoR and rpoF were used to detect *Bartonella* spp. These primers (1400F and 2300R) amplify an 850 bp fragment of the *rpoB* gene [35]. The PCR test was conducted according to Renesto et al. [35]. The PCR reactions were conducted in a 50 µl reaction mixture containing 2 µl of DNA template, 0.5 U (0.1 µl) of RUN Taq polymerase (A&A Biotechnology, Gdynia, Poland), 1 µl of dNTPs (10 mM), 0.5 µl of each primer (20 mM), and 5 µl of 10 × Taq DNA polymerase buffer (pH 8.6, 25 mM MgCl_2_). In the negative control, nuclease-free water was added to the PCR mix instead of the tested DNA. Both reactions were performed using the DNA Engine PTC-200 Thermal Cycler (BioRad, Hercules, USA) according to the following program: initial denaturation was performed at 94 °C for 5 min, followed by 35 cycles of denaturation at 95 °C for 10 s, annealing at 60 °C for 10 s and extension at 72 °C for 60 s. The final extension was performed at 72 °C for 7 min and then kept at 10 °C. The PCR products were visualized on a 1.0% agarose gel stained with ethidium bromide. Visualization was performed using ChemiDoc, MP Lab software (Imagine, BioRad, Hercules, USA). The resulting product was compared using the Nova 100 bp DNA Ladder Novazym (Poznań, Poland). The PCR amplicons were purified using a QIAEX II Gel Extraction Kit (Qiagen, Hilden, Germany), sequenced in both directions by Genomed (Poland), and contiguous sequences assembled using ContigExpress, Vector NTI Advance 11.0 (Invitrogen Life Technologies, New York, USA). The derived sequences were submitted to the GenBank database under the accession numbers MH669401-MH669404. To the phylogenetic studies, there was used Bayesian inference (BI) analysis with MrBayes version 3.2 [17]. Analysis of partial rpoB gene sequence data was based on an alignment of 762 bp (254 amino acids) using GTR + I + G model. The GTR models were chosen on the basis of jModelTest version 2.1.4 [10, 14] using Akaike information criterion.

## Results

**Fig. 1:**
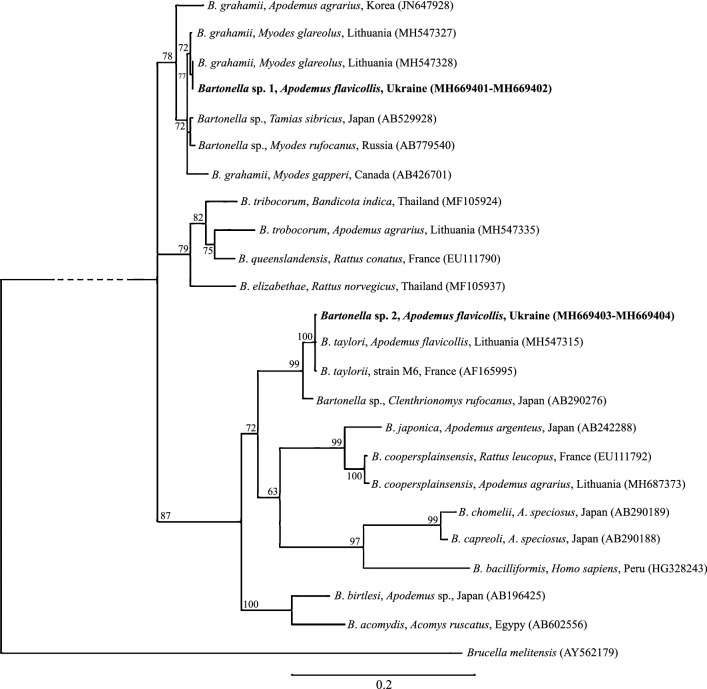
Phylogenetic tree of *Bartonella* spp., constructed by Bayesian inference (BI) analysis using MrBayes version 3.2. For BI codon analysis (nucmodel = codon), the GTR + I + G model was chosen based on jModelTest version 2.1.4 [10, 14] using Akaike Information Criterion. Analysis was run for 8,000,000 generations, with 2,000,000 generations discarded as ‘burn-in’. Hosts, country and GenBank accession numbers of origin are shown. Nodal support is indicated as Bayesian posterior probabilities. Sequence from *Brucella melitensis* (AY562179) was used as outgroup. Sequences generated in this study are show in bold

The molecular analysis revealed a higher prevalence of *Bartonella* spp. infection, with the total infection being 38.89% (14/36). In *Apodemus flavicollis*, the prevalence was 75% (9/12). The prevalence in *Microtus arvalis* (33.3%, 1/3) and *Apodemus agrarius* (28.57%, 2/7) was similar while the prevalence in *Myodes glareolus* was 15.38% (2/13). No infected individuals were found in the case of *Apodemus sylvaticus*. Two sequences (*Bartonella* sp. 1: MH669401-MH669402) obtained in this study share 100% similarity with *B*. *taylorii* isolated from *Apodemus flavicollis* from Lithuania (GenBank: MH547315) and *B*. *taylorii* strain M6 from France (GenBank: AF165995). In turn, two sequences (*Bartonella* sp. 2: MH669403-MH669404) showed 100% similarity with *B*. *grahamii* isolated from *Myodes glareolus* from Lithuania (MH547328) and 98.1% similarity with *Bartonella* sp. isolated from *Clethrionomys rufocanus* from Japan (AB290276) (Table 1; Fig. 1).

**Table 1:** *Bartonella* spp. used in the phylogenetic analysis

*Bartonella* isolates	Species	Host	Country of isolation
Apf10, Apf18	*Bartonella* sp. 1	*Apodemus flavicollis*	Ukraine
Apf34, Apf9	*Bartonella* sp. 2	*Apodemus flavicollis*	Ukraine
AB602556	*Bartonella acomydis*	*Acomys ruscatus*	Egypt
AB196425	*Bartonella birtlesi*	*Apodemus* sp.	Japan
HG328243	*Bartonella bacilliformis*	*Homo sapiens*	Peru
AB290189	*Bartonella chomelii*	*Apodemus speciosus*	Japan
AB290188	*Bartonella capreoli*	*Apodemus speciosus*	Japan
MH687373	*Bartonella coopersplainsensis*	*Apodemus agrarius*	Lithuania
EU111792	*Bartonella coopersplainsensis*	*Rattus leucopus*	France
AB242288	*Bartonella japonica*	*Apodemus argenteus*	Japan
AB290276	*Bartonella* sp*.*	*Clethrionomys rufocanus*	Japan
AF165995	*Bartonella taylorii*	strain M6	France
MH547315	*Bartonella taylorii*	*Apodemus flavicollis*	Lithuania
MF105937	*Bartonella elizabethae*	*Rattus norvegicus*	Thailand
EU111790	*Bartonella queenslandensis*	*Rattus conatus*	France
MH547335	*Bartonella tribocorum*	*Apodemus agrarius*	Lithuania
MF105924	*Bartonella tribocorum*	*Bandicota indica*	Thailand
AB426701	*Bartonella grahamii*	*Myodes gapperi*	Canada
AB779540	*Bartonella* sp.	*Myodes rufocanus*	Russia
AB529928	*Bartonella* sp.	*Tamias sibiricus*	Japan
MH547328	*Bartonella grahamii*	*Myodes glareolus*	Lithuania
MH547327	*Bartonella grahamii*	*Myodes glareolus*	Lithuania
JN647928	*Bartonella grahamii*	*Apodemus agrarius*	Korea

## Discussion

Our findings indicate the presence of *Bartonella* species in rodents inhabited Chernobyl Exclusion Zone in Ukraine. The nearest studies of *Bartonella* infection in rodents have been conducted in Poland, Slovakia and Lithuania. In all of these countries, rodents were tested for bacteria genus *Bartonella*. The prevalence in all animals was 38.89% and it is comparable with results from Poland (22.4%) [33], and lower in Slovakia (64.8%) [38]. The prevalence among rodent species was varied. The prevalence in *Apodemus flavicollis* was high (75%), and was similar in another countries: Poland (66.6%) [18], Slovakia (63%) [38] and Lithuania (80%) [27]. The obtained prevalence of this bacterium in *Myodes glareolus* was 33.3%. This result was identical in Lithuania [27] and similar with result obtained in Poland (38.7% in the Mazurian Lakeland, 33.3% in Białowieża Forest) [4, 18] or from southwestern Slovakia (69.0%) [38]; however, they are higher than in western Slovakia (9.7%) [36]. In the case of *Microtus arvalis*, the prevalence of *Bartonella* spp. infection was 33.3%. This value is comparable with that obtained previously from Poland (27.7%) by Pawełczyk et al. [32], and in a PCR study in Mazuria, Poland (33.3%) by Paziewska et al. [33]. In contrast, this value was markedly higher than another result from Central Europe, where Šebek [36] identified a prevalence of 7.7% in western Slovakia. Compared to other rodent species, *Apodemus agrarius* has been poorly studied. Our studies show prevalence higher (28.57%) than in Slovakia [24] and Lithuania [27]. No infected individuals were found in the case of *Apodemus sylvaticus*, but we obtained only one animal. Two sequences (*Bartonella* sp. 1) obtained in this study are identical with *B*. *taylorii* isolated from *Apodemus flavicollis* from Lithuania (GenBank: MH547315) and *B*. *taylorii* strain M6 from France (GenBank: AF165995). This suggests that this *Bartonella* species occurs throughout Europe. Two sequences (*Bartonella* sp. 2) are identical with *B*. *grahamii* isolated from *Myodes glareolus* from Lithuania (MH547328) and 98.1% similarity with *Bartonella* sp. isolated from *Clethrionomys rufocanus* from Japan (AB290276). There is the first evidence of the infection of rodents in Chernobyl Exclusion Zone with *Bartonella* spp. The similarity of obtained *rpoB* gene sequences to isolates from Lithuania, as well from France, published in GenBank, indicates that rodents in CEZ are infected with common species of *Bartonella*. The follow-up studies are necessary to obtain the number of samples enough to statistical analysis, as well study conducted whole year, to describe the seasonal dynamic.

Our findings indicate that rodents in the Chernobyl Exclusion Zone are infected by strains of *Bartonella* common in Europe. The study presents the first record of bacteria genus *Bartonella* in rodents in the Chernobyl Exclusion Zone.
